# The associations between child behavioral problems, parents’ emotional regulation difficulties, and parental burnout among Israeli parents of children with autism during wartime

**DOI:** 10.3389/fpsyg.2024.1439384

**Published:** 2024-11-25

**Authors:** Shlomit Shnitzer-Meirovich, Shani Aviad, Inbal Bechar-katz, Tal Araten-Bergman, Vered Shenaar-Golan, Ayelet Gur

**Affiliations:** ^1^The Academic College Levinski-Wingate, Tel-Aviv, Israel; ^2^School of Allied Health, Human Services and Sports, and Living with Disability Research Centre College of Science, Health & Engineering, La Trobe University, Melbourne, VIC, Australia; ^3^Social Work Department, Faculty of Social Sciences & Humanities, Tel-Hai College, Qiryat Shemona, Israel; ^4^Child and Adolescent Mental Health Clinic, Ziv Medical Center, Safed, Israel; ^5^Research Center for Innovation in Social Work, Faculty of Social Sciences & Humanities, Tel-Hai College, Qiryat Shemona, Israel

**Keywords:** parental burnout, emotion regulation, behavioral problems, ASD, war

## Abstract

**Introduction:**

Parents of children with Autism Spectrum Disorder (ASD) face heightened challenges during crises like war, leading to parental burnout (PB). Wartime demands may exacerbate the children’s behavioral difficulties, which associated with PB. Successful emotional regulation (ER) is considered a protective factor for PB. This study aimed to explore the associations between the child behavioral problems, parent’s ER difficulties and PB among parents of children ASD during wartime.

**Methods:**

92 parents completed demographic, Parental Burnout, Difficulties in Emotion Regulation, and Child Aberrant Behavior questionnaires. In addition, 18 parents of children with ASD aged 6–21 were semi-interviewed.

**Results:**

During wartime, child behavioral problems and parents’ ER difficulties were positively correlated with PB, contributing beyond background characteristics and other changes following the war. Parent ER difficulties serve as a mediation variable and did not function as a moderation variable between child behavioral problems and PB.

**Discussion:**

This study emphasizes the increased vulnerability of parents of children with ASD during wartime, highlighting the need for a deeper understanding of how these circumstances affect parental risk and resources. Effective interventions should target emotional regulation and address child behavioral issues, necessitating prioritized support for affected families despite wartime challenges.

## Introduction

Autism spectrum disorder (ASD) is a neurodevelopmental disorder which emerges in early childhood, characterized by persistent deficits in both (1) social communication and interaction, and (2) restricted and repetitive behaviors (American Psychological Association, 2022). In addition to these core symptoms, children with ASD frequently exhibit emotional and behavioral issues such as hyperactivity, aggression, tantrums, refusing to participate in daily activities, disruptive behavior, and self-harming conduct (Kaba et al., 2023; Yılmaz et al., 2021). In March 2022, approximately 1 in 100 children was diagnosed with ASD (World Health Organization, 2022), and the prevalence of ASD continues to grow (Salari et al., 2022).

Children diagnosed with ASD face difficulties in their daily routine lives. In times of emergency, such as wartime, resulting in the closure of schools, support services, and community programs, these children are at an increased risk for experiencing negative outcomes. Since children with ASD are extremely sensitive to changes in their routine, the disruption of critical services and therapies, accompanied by other changes in their day to day lives, may lead to the worsening of their symptoms, increased behavioral and emotional challenges, and decreased mental well-being (White et al., 2021).

Parents of children with ASD often experience emotional and informational gaps. They tend to struggle with depression, confusion, increased stress and anxiety, loneliness, labeling, and face marital problems (Koçak et al., 2023). During times of crisis, the exacerbated behavioral difficulties in children with ASD may aggravate the parents’ mental health, due to the tenacious link between a child’s emotional and behavioral problems and their parent’s mental wellbeing (Sekułowicz et al., 2022; Yılmaz et al., 2021; Yorke et al., 2018). As a result, during wartime, parents of children with ASD are particularly vulnerable to burnout (Sekułowicz et al., 2022).

Parental burnout (PB), defined as chronic physical and mental fatigue (Burisch, 2006), is a chronic stress-related syndrome experienced in the parental role. It denotes chronic suffering that occurs when parents’ resources are unable to compensate for their parenting stress over a chronic period of at least three months (Lin et al., 2022). PB encompasses four dimensions: physical/emotional exhaustion, emotional distancing from children, feeling overwhelmed by parenting duties, and contrast between current and past parenting experiences (Ardıc and Olçay, 2021; Findling et al., 2023; Lin et al., 2022; Lin et al., 2023; Mikolajczak and Roskam, 2018; Mikolajczak et al., 2019).

Research shows that PB is directly affiliated with the child’s particular characteristics, as they determine the intensity of the demands on the parents and may lead to an increased risk for burnout, especially regarding a child with a disability, a chronic disease or behavioral or emotional disorders (Sekułowicz et al., 2022). In their study, Kaba et al. (2023) assert that there is a stronger link between PB and a child’s behavioral problems than the frequency and severity of ASD symptoms. This finding shifts the focal point from examining the correlation between the severity of a child’s autism and PB to investigating the relationship between the child’s behavioral issues, which appear to exert a robust and significant influence that deserves further scrutiny and attention.

The Balance between Risks and Resources (BR2) theory presented by Mikolajczak and Roskam (2018) defines PB as a context-specific syndrome that develops among parents with chronic parenting stress, and results from an imbalance between the demands of the parenting role and the resources a parent requires to cope. The theory posits that being a parent to a child with ASD, along with the substantial responsibility of tending to a child with developmental and behavioral challenges, might be seen as an inherent risk factor.

To the best of our knowledge, there have not been any studies specifically examining PB among parents of children with ASD in times of war. However, research conducted during other emergencies, such as the Covid-19 pandemic, shares comparable traits and similarities that could offer insights. Research conducted during the Covid-19 pandemic indicated that the sudden changes to routines enforced by the circumstances of the pandemic, led to exacerbated behavioral difficulties in children with ASD, which predicted parental stress. The difficulty parents faced in managing these increased behavioral issues have strained parents’ abilities to cope with their stress (Bentenuto et al., 2021; Kaba et al., 2023; Manning et al., 2021; Mutluer et al., 2020). Moreover, studies examining PB among parents of children with ASD during Covid-19 found that they experienced higher levels of PB compared to parents of children in the control groups (Kaba et al., 2023; Kütük et al., 2021; Yılmaz et al., 2021) as well as parents of children with other special needs (Çimen et al., 2023). The parents’ sense of burden of care leads them to face a variety of unique difficulties that harm their physical health and mental well-being (Findling et al., 2023).

It therefore follows that during wartime, it is extremely essential to identify the resources/protection factors that can help parents to compensate for the burdensome weight of wartime demands. Taking into consideration that parents cannot negate the demands imposed by war, they can find a way to assess the impact of those demands on their lives by increasing internal resources. One example of such internal resource is high Emotion regulation (ER) skills.

ER refers to individuals exerting control over which emotions they feel, the timing of these emotions, and how they perceive and display them (Gross, 1998). Efficient ER means the individual can (a) evaluate contextual demands, (b) possess a repertoire of strategies from which they can choose the most appropriate one, and (c) monitor the efficacy of that strategy (in terms of its outcomes) and modify the strategy if needed (Bonanno and Burton, 2013; Brandão et al., 2024). Unlike personality attributes, ER strategies are skills that can be taught and learned during therapy, thereby impact people’s emotions in meaningful ways and reduce symptoms (Hallion et al., 2018; Wang et al., 2021).

Adaptive ER strategies are frequently linked to positive outcomes and are considered as protective factors for the development of psychopathology (Aldao et al., 2010; Lincoln et al., 2022). On the contrary, maladaptive ER strategies are considered risk factors for the development of psychopathology and are associated with negative outcomes such as poor well-being and poor interpersonal relationship outcomes (McRae and Gross, 2020).

To date, prior research has indicated that the higher the parents’ sense of burden of caring for the child is, the more negative the emotions the parents experience when caring for their child and vice versa (Findling et al., 2023; Hajal and Paley, 2020). According to the literature, the ability of primary caregivers to experience and manage emotions is related to the sense of stress they experience and their perception of the burden of care (Kim and Kim, 2015). Importantly, higher levels of emotional control were found to be associated with higher levels of positive parenting and lower levels of harsh parenting (Hajal and Paley, 2020).

Being a parent is considered one of the most emotion-abundant contexts in one’s life, and parents of children with ASD experience even more emotionally related risk factors compared to parents of children with typical development (Kerr et al., 2021; Lin et al., 2021). ER development for parents of children with special needs is extremely important since the behavioral and emotional problems of these children evoke significant challenges among their parents who already face emotional strains (Prikhidko and Swank, 2019). Despite the importance of ER, parents are rarely educated about it and there is limited research available on ER strategies that parents in general, and parents of children with ASD in particular, implement to regulate negative emotions (Prikhidko and Swank, 2019).

Efficient ER strategies have proven to be a possible internal resource that aids in reducing PB (Brandão et al., 2024). One possible explanation to this relation is that burnout is caused not by emotions and activities as such, but rather by excessive effort invested in regulating them during continuous and adverse circumstances (Kwiatkowski and Sekulowicz, 2017). According to the BR2 theory (Mikolajczak and Roskam, 2018), difficulties in ER are considered to be a transdiagnostic risk for PB, while successful ER is considered a protective factor for PB. High ER skills may help to significantly decrease parental stress leading to a reduction in PB. Indeed, a systematic review and meta-analysis of the relationship between ER and PB has found that expressive suppression, meaning individuals’ attempts to conceal, avoid, or reduce the emotion expressive behavior (Gross and Levenson, 1993) was positively related to PB, whereas cognitive reappraisal, i.e., changing emotional experiences by thinking differently about a situation (John and Gross, 2004) was negatively related to PB (Brandão et al., 2024). It therefore seems that efforts to inhibit emotional experiences contribute to higher difficulties in dealing with parenting stress, which potentially lead to PB.

The association of reappraisal with PB may be due (in part) to the role of ER skill as acting as a protective factor buffering the impact of risk factors related to children’s variables (e.g., difficult temperament, disability, behavioral problems) on PB (Lin et al., 2022; Swit and Breen, 2023). It appears that efficient ER strategies contribute to coping with parenting demands and moderating their harmful effects, even in the presence of other constraints such as lack of social support and stress (Brandão et al., 2024; Rodriguez et al., 2020).

Another assumption is that children’s behavioral and emotional difficulties interrupt the parents’ ability to use adaptive ER strategies, leading them to experience higher degree of burnout. As argued by Bosse et al. (2010), it is not plausible to have one set of ‘ideal’, innate regulation characteristics applicable in various contexts. Instead, emotion regulation should be viewed as an adaptive process, where the flexibility to choose different regulatory strategies can be adapted based on an assessment of the emotion regulation process’s effectiveness in specific situations. According to Hajal and Paley (2020), parents’ behaviors are fundamentally shaped by their emotional experiences, and when faced with children’s emotional needs, the parenting situation becomes particularly emotionally evocative. They note that parent–child interactions can become mutually dysregulating, creating cyclical patterns. A recent study found that the child’s emotional regulation serves as a mediating factor between their behavioral problems and parental functioning (Qiao et al., 2024). Considering the close relationship between the child’s emotional regulation and that of the parent, it can be hypothesized that the parent’s emotional regulation would also act as a mediating factor between the child’s behavioral problems and aspects of parental functioning, and similarly, on PB.

This theoretical framework suggests a potential mediational pathway where emotion regulation could serve as the mechanism through which child behavior challenges influence parental outcomes. Since parenting inherently triggers strong emotions that require regulation, and the ability to regulate these emotions is linked to parenting quality, emotion regulation may be a critical intervening variable in determining how parents respond to and cope with challenging child behaviors.

Examining these relations during emergency times has additional significance. Studies examining the role of ER on PB conducted during the Covid-19 pandemic found that ER was a significant moderator of the link between the negative effects of Covid-19 and PB (Prikhidko et al., 2020; Swit and Breen, 2023; Vertsberger et al., 2022). Another study showed a strong link between parents’ ER strategies and PB during the pandemic (Santelices et al., 2022).

Overall, it seems that the pattern of association between ER and PB was consistent across the context (COVID vs. no COVID) (Brandão et al., 2024). However, none of the previous studies were conducted among parents of children with special needs. Furthermore, as far as we know, no study to date has examined these relations during wartime.

The present study was conducted during the “Swords of Iron” war between Israel and Hamas. As the war broke out, all state services in Israel were disrupted, disturbed, or suspended, including welfare, health, economic, transportation, and particularly educational services. The entire education system was shut down for several weeks. In addition, many Israeli citizens were forced to leave their homes for an extended period due to safety concerns. These unusual circumstances had an additional impact on children with ASD and their parents. In order to better understand the potential benefit of ER of parents of children with ASD to this population during emergency times, the goal of the present study is to examine the role of ER in the relationship between a child’s behavioral problems and PB among parents of children with ASD during wartime.

Since there is a lack of research addressing parental stress in emergencies, particularly during wartime among parents of children with ASD, the current study poses 4 quantitative research hypotheses and one qualitative research question:

1. Child’s behavioral problems and parent ER difficulties correlate with PB during the time of war among parents of children with ASD, as previous studies show these correlations during routine.
2. Parents’ and children’s background characteristics (e.g., gender, education level, marital status), as well as changes in the family routine, have a significant contribution in explaining the child’s behavioral problems, parent ER difficulties, and PB during the Swords of Iron war.
3. Child behavioral problems and parent ER difficulties have a unique contribution beyond parents’ and children’s background characteristics and changes in the family routine, to PB during the Swords of Iron war among parents of children with ASD.
4. Parent ER difficulties play as a moderator and/or a mediator in the association between child behavioral problems and PB during the Swords of Iron war among parents of children with ASD.

*Qualitative question*: How do parents of children with ASD perceive their coping during wartime?

## Method

### Study design

The study employed a mixed methods approach, combining both quantitative and qualitative components. The quantitative component involved an online survey administered to 92 parents of children with ASD during the war. Complementing this, the qualitative component consisted of in-depth interviews with 18 parents from the same population.

### Participants

The current study comprised of 92 parents (15 males and 77 females) aged 33 to 61 (*M* = 44.50, *SD* = 5.65). All parents have between 1 to 5 children (*M* = 2.47, *SD* = 0.93) while at least one of them diagnosed with ASD. The ASD diagnosis of all children was made by a clinical psychologist, developmental physician, neurologist, or child psychiatrist. The children’s ages ranged from 4 to 21 years old (*M* = 9.41, *SD* = 3.63). Five parents had more than 1 child with ASD (5.4%). One parent indicated that both children who diagnosed with ASD are girls and the other four parents indicated that they have both genders diagnosed with ASD. The parents were asked several questions regarding their background characteristics and their children as well as several questions regarding the changes in work, residence, main caregiver etc. during the Swords of Iron war (see Table 1). Table 2 presents the sample characteristics of the qualitative component.

**Table 1:** Parent’s and children’s background characteristics (*N* = 92).

Background characteristics	Values	Frequency (%)
Parent’s gender	Male	15 (16.3%)
	Female	77 (83.7%)
Education	High school	12 (13.0%)
	Professional certificate	13 (14.1%)
	B.A.	36 (39.1%)
	M.A.	27 (29.3%)
	PhD.	4 (4.3%)
Marital status	Married/shared parenting	72 (78.3%)
	Divorced/separated	10 (10.9%)
	Single parent	10 (10.9%)
Change in work following the war	No	64 (69.6%)
	Yes	28 (30.4%)
Change in residence following the war	No	86 (93.5%)
	Yes	6 (6.5%)
A change in the main caregiver of the child(ren) following the war	No	84 (91.3%)
	Yes	8 (8.7%)
Change in the educational setting of the child(ren) following the war	No	50 (54.3%)
	Yes	42 (45.7%)
Change in supports, treatments, or routine classes of the child(ren)	No	47 (51.1%)
	Yes	45 (48.9%)
A family member or close acquaintance who was injured/killed in the war	No	79 (85.9%)
	Yes	13 (14.1%)
A family member or close acquaintance who serves in the army	No	46 (50.0%)
	Yes	46 (50.0%)
Child’s gender	Boy	71 (77.2%)
	Girl	17 (18.5%)
	Both genders	4 (4.3%)

**Table 2:** Sample characteristics of the qualitative component (*N* = 18).

Interviewee	Gender	Age	Status	Child’s age	Level of functioning	Residence
1	Man	52	Married	9.5	High	center of Israel
2	Man	46	Married	14.5	Moderate- High	center of Israel
3	Man	44	Married	10	High	center of Israel
4	Woman	47	Married	12.5	High	center of Israel
5	Man	45	Married	11	High	center of Israel
6	Woman	40	Married	9	Moderate-High	center of Israel
7	Man	40	Married	2.5	Low	center of Israel
8	Woman	35	Married	6	High	center of Israel
9	Woman	45	Married	6	Moderate- Low	North of Israel
10	Woman	45	Married	6	Moderate- High	center of Israel
11	Woman	41	Married	5	Moderate- High	center of Israel
12	Woman	40	Married	4	Low	center of Israel
13	Woman	34	Married	4.5	Moderate- Low	center of Israel
14	Woman	45	Single	4.5	Moderate- High	center of Israel
15	Woman	29	Married	4	Low	North of Israel
16	Woman	35	Divorced	5	Moderate- Low	North of Israel
17	Woman	36	Married	3	Moderate	center of Israel
18	Woman	32	Married	4	Moderate	North of Israel

### Materials

#### The Aberrant Behavior Checklist (ABC)

Aman et al. (1985) is an empirically constructed measure of the problem behaviors on which caregivers’ rate 58 items on a 4-point Likert scale of severity, ranging from zero (not at all a problem) to three (the problem is severe in degree). The original tool consists of five sub-scales (irritability, social withdrawal, stereotypy, hyperactivity, and inappropriate speech). In our study, we used only the irritability sub-scale (15-item), and the hyperactivity/noncompliance subscale (16-item). A large analysis of *N* = 1,893 youth with ASD conducted by Kaat et al. (2014) has found that of the ABC dimensions, the irritability subscale explains the most variation in parent report of problem behaviors and that as previously reported (Aman et al., 1985) the correlations were highest between the irritability and hyperactivity/noncompliance subscales. The internal consistency of Cronbach alpha was high for the 31 items (*α* = 0.97) and for both irritability (*α* = 0.94) and hyperactivity/noncompliance (*α* = 0.95) subscales.

#### The Parental Burnout Assessment (PBA)

PB was measured using the PBA (Roskam et al., 2018) which includes 23 items. The questionnaire was translated from English-to-Hebrew using the translation–back-translation procedure (Findling et al., 2023). It includes four PB dimensions: Physical and emotional exhaustion in the parental role (9 items); Emotional distancing from the child (3 items); Saturation from the parental-role (5 items); and Contrast with previous parental-self (6 items). Parents were asked to indicate how often they felt each emotion since October 7th (the 1st day of war) on a scale ranging from 1 = “not at all” to 7 = “every day.” The internal consistency of Cronbach’s alpha for all 23 items was high *α* = 0.97 as well as for each of the four dimensions [α = 0.93 for physical and emotional exhaustion, α = 0.86 for emotional distancing, α = 0.90 for Saturation and α = 0.93 for contrast with previous parental-self].

#### Difficulties in Emotion Regulation Scale (DERS)

The DERS (Gratz and Roemer, 2004) is considered a comprehensive, global measure of difficulties in ER with the aim to assess multidomain (i.e., cognitive, affective, behavioral) aspects of emotion dysregulation (Sörman et al., 2022). It includes 36 items and six subscales. Each item is scored on a 5-point Likert scale, ranging from 1 (almost never) to 5 (almost always), with higher scores reflecting more difficulties in ER (i.e., total score ranging from 36 to 180). The 36 items self-report scale asks respondents how they relate to their emotions in order to produce scores on the following subscales: Nonacceptance (reflecting nonacceptance of emotional responses, 6 items); Goals (reflecting difficulties engaging in goal-directed behavior in stressful situations, 5 items); Impulse (reflecting impaired ability to control impulsive behaviors when distressed, 6 items); Awareness (reflecting lack of emotional awareness, 6 items); Strategies (reflecting limited access to different ER-strategies, 8 items); and Clarity (reflecting lack of emotional clarity, 5 items). The internal consistency of Cronbach’s alpha for all 36 items was high α = 0.94 as well as for each of the six subscales [α = 0.89 for nonacceptance, α = 0.87 for goals, α = 0.90 for impulse, α = 0.83 for awareness, α = 0.89 for strategies and α = 0.74 for Clarity].

#### Qualitative interview guide

The interviews commenced with a broad, open-ended question, inviting participants to provide a comprehensive overview of their parenting experiences with their child. Following this general inquiry, more targeted and specific questions were posed to delve deeper into the participants’ experiences during wartime.

The interview began with an open-ended question: “Could you tell me about your experience as a parent of a child with ASD?”

After participants shared their parenting journey, several follow-up questions were explored. Parents were invited to reflect on how the war had affected them emotionally, and to describe the unique challenges they faced in caring for their child with ASD during this time. The discussion encompassed their general emotional state, self-perception, and the dynamics within their family unit.

The conversation then turned to how parents viewed their parental role during this crisis period. Finally, participants were asked to share their sources of strength and the factors that helped them maintain resilience during these challenging times.

### Procedure

The research proposal was submitted to obtain ethics approval from the Institutional Ethics Committee of the Levinsky-Wingate Academic Center (protocol number: 2024013001). The sampling method of the parents was purposive sampling. In this sampling method, the subjects are purposefully selected based on the characteristics suitable for the study (Etikan et al., 2016). The questionnaire was administered via social networks and forums of parents of children with ASD. Parents provided informed consent and were assured of anonymity. They could withdraw from the study at any time. For qualitative interviews, participants shared their contact information and consented to be interviewed. The interviews were scheduled via Zoom at a time convenient for the interviewee. The length of the interview was about an hour and was predetermined for the interviewees.

At this phase, parents also had the option to discontinue their participation in the research at any time.

### Data analyses

In order to examine the correlations between the child behavioral problems, parent ER difficulties and PB during the Swords of Iron war, Pearson correlation analyses were conducted. To examine the contribution of parent’s and children’s background characteristics as well as the changes in the family routine to the child behavioral problems, parent ER difficulties and PB during the Swords of Iron war, three multiple regression analyses were conducted, one analysis for each measure. To examine the unique contribution of child behavioral problems and parent ER beyond the parent’s and children’s background characteristics and the changes in the family routine to the PB during the Swords of Iron war, hierarchical regression analysis was conducted. Finally, to examine the role of the parent ER difficulties in the association between child behavioral problems and PB during the Swords of Iron war, moderation analysis using model 1 and mediation analysis using model 4 were conducted using the PROCESS software (Hayes, 2018).

The qualitative data collected from the in-depth interviews were analyzed using thematic and categorical approaches aimed at identifying recurring patterns, themes and categories relevant to the research question. Initially, the transcripts were read and reread to develop familiarity with the data. Next, a coding process was used to systematically label and categorize text fragments representing significant concepts, experiences, and perspectives expressed by participants in relation to the research question. The coding was done by marking quotes and themes that were repeated among the majority of participants in the study, finding a common denominator, and writing the interpretation according to the categories that came up. Next, triangulation was used to increase the trustworthiness and reliability of the findings. The triangulation involved combining the qualitative data with two additional sources of information: quantitative data obtained from the online questionnaire and relevant theoretical frameworks related to the research topic. The qualitative part provides an explanation for the quantitative results as the participants in the study reported in the interviews.

## Results

Descriptive statistics of the child behavioral problems, parent ER difficulties and PB are presented in Table 3.

**Table 3:** Descriptive statistics of the child behavioral problems, parent ER difficulties and PB during the Swords of Iron war (*N* = 92).

Scale	Sub-scale	*M*	*SD*	Min-Max
Child behavioral problems (Range 0–3)	Total scale	0.95	0.72	0.00–2.94
	Irritability	0.90	0.74	0.00–2.87
	Hyperactivity/Noncompliance	1.00	0.76	0.00–3.00
Parent ER difficulties (Range 1–5)	Total scale	2.45	0.69	1.14–4.11
	Nonacceptance of emotional responses	2.60	1.07	1.00–5.00
	Difficulty engaging in goal-directed behavior	2.73	0.99	1.00–4.80
	Impulse control difficulties	2.32	1.03	1.00–5.00
	Lack of emotional awareness	2.67	0.87	1.00–4.67
	Limited access to emotion regulation strategies	2.39	0.90	1.00–4.25
	Lack of emotional clarity	1.97	0.73	1.00–3.60
PB (Range 1–6)	Total scale	2.16	1.12	1.00–5.52
	Exhaustion in parental role	2.58	1.26	1.00–5.56
	Emotional distancing	1.79	1.13	1.00–5.67
	Feelings of being fed up	1.75	1.07	1.00–5.20
	Contrast in parental self	2.05	1.24	1.00–5.83

As can be seen in Table 3, the parents of children with ASD indicated medium-low level of child behavioral problems, parent ER difficulties and PB are in relation to the original scales of these measures during the Swords of Iron war.

### Q1: correlation between the child behavioral problems, parent ER difficulties, and PB during the Swords of Iron war

In order to examine the first research question, Pearson correlation analyses were conducted. The results indicated that the child behavioral problems were positively correlated with PB [*r*(90) = 0.54, *p* < 0.001] as well as the positive correlation that was found between parent ER difficulties with PB [*r*(90) = 0.59, *p* < 0.001]. In addition, the child behavioral problems measure was positively correlated with parent ER difficulties measure [*r*(90) = 0.40, *p* < 0.001]. These results indicated that higher behavioral problems of the child with ASD and higher parent ER difficulties associate with higher PB during the war.

### Q2: contribution of parent’s and children’s background characteristics as well as the changes in the family routine to the child behavioral problems, parent ER difficulties and PB during the Swords of Iron war

In order to examine the second research question, three multiple regression analyses were conducted, one for each study measure. The explanatory variables in the model were entered in a stepwise manner. According to this manner, only variables that contribute significantly to the Explained Variance (EPV) of the child behavioral problems, parent ER difficulties and PB during the Swords of Iron war were entered into the model. The variables were entered in accordance with the level of their contribution significance.

The results indicated that the change in the educational setting of the child(ren) following the war increase the child behavioral problems (*R^2^* = 15.2%), parent ER difficulties (*R^2^* = 8.1%) and PB (*R^2^* = 21.8%). Moreover, this is the first variable which was entered into the regression model in all three analyses. In addition, regarding the level of PB, the change in the main caregiver of the child(ren) following the war contributed additionally 4% to the EPV, beyond the educational setting change. Finally, the child’s gender contributed additionally 4% to the EPV of the child behavioral problems, beyond the educational setting change. The positive *β* coefficient indicating that parents of boys with ASD tent to declare higher child behavioral problems during the war (see Table 4).

**Table 4:** Results of the multiple regression analyses for the child behavioral problems, parent ER difficulties and PB during the Swords of Iron war (*N* = 92).

Explained variables	Explanatory variables	*B*	*SE.B*	*β*	*R^2^*	*∆R^2^*
Child behavioral problems	Change in educational setting^1^	0.56	0.14	0.40***	0.152***	----
	Child’s gender^2^	0.36	0.17	0.20*	0.193***	0.041*
ER difficulties	Change in educational setting^1^	0.39	0.14	0.29**	0.081**	----
PB	Change in educational setting^1^	0.96	0.20	0.45***	0.218***	----
	Change in main caregiver^3^	0.76	0.35	0.20*	0.258***	0.040*

*p < 0.05, **p < 0.01, ***p < 0.001; ^1^Change in educational setting: 0 = No, 1 = Yes; ^2^Child’s gender: 0 = Boy, 1 = Girl; ^3^Change in main caregiver: 0 = No, 1 = Yes.

### Q3: contribution of child behavioral problems and parent ER beyond the parent’s and children’s background characteristics and the changes in the family routine to the PB during the Swords of Iron war

In order to examine the third research question, hierarchical regression analysis was conducted. The explanatory variables in the model were entered in three blocks. In the first block, the background characteristics as well as the changes in the family routine were entered in a stepwise manner. In the second block, the child behavioral problems measure was entered, to examine the unique contribution of this scale beyond the background characteristics. In the third block, the parent ER difficulties measure was entered to examine the unique contribution of this scale beyond the background characteristics and beyond the child behavioral problems.

The results of the regression analyses indicated that the child behavioral problems contributed 13.6% beyond the first block to the EPV of PB. The positive β coefficient indicating that the higher the child behavioral problems are, the higher the PB is. In addition, the parent ER difficulties contributed 11% beyond the first and the second blocks to the EPV of PB. The positive β coefficient indicating that the higher the parent ER difficulties are, the higher the PB is (see Table 5).

**Table 5:** Results of the hierarchical regression analyses for the PB by the parent’s and children’s background characteristics, child behavioral problems and parent ER difficulties.

Blocks	Explanatory variables	*B*	*SE.B*	*β*	*R^2^*	*∆R^2^*
1	Change in educational setting^1^	0.96	0.20	0.45***	0.218***	----
	Change in main caregiver^2^	0.76	0.35	0.20*	0.258***	0.040*
2	Change in educational setting^1^	0.62	0.20	0.29**		
	Change in main caregiver^2^	0.84	0.32	0.22**		
	Child behavioral problems	0.62	0.14	0.40***	0.394***	0.136***
3	Change in educational setting^1^	0.51	0.18	0.24**		
	Change in main caregiver^2^	0.57	0.30	0.15		
	Child behavioral problems	0.44	0.14	0.28**		
	ER difficulties	0.58	0.14	0.37***	0.504***	0.110***

*p < 0.05, **p < 0.01, ***p < 0.001; ^1^Change in educational setting: 0 = No, 1 = Yes; ^2^Change in main caregiver: 0 = No, 1 = Yes.

### Q4: the role of the parent ER difficulties in the association between child behavioral problems and PB during the Swords of Iron war

In order to examine the fourth research question, moderation analysis using model 1 and mediation analysis using model 4 in PROCESS software (Hayes, 2018) were conducted. The variables: the change in the educational setting and main caregiver of the child(ren) following the war were taken as covariate variables in the moderation and mediation analyses, since these two variables contributed significantly to the EPV of PB.

#### Moderation analysis

The results indicated that the parent ER difficulties did not serve as a moderation variable between child behavioral problems and PB during the Swords of Iron war. Meaning that the interaction between the child behavior problem and parent ER difficulties is not significant (*B* = 0.19, *SE* = 0.19, *LLCI*(95%) = −0.20, *ULCI*(95%) = 0.57, *∆R^2^* = 0.53%, *p* = 0.336).

#### Mediation analysis

**Figure 1:**
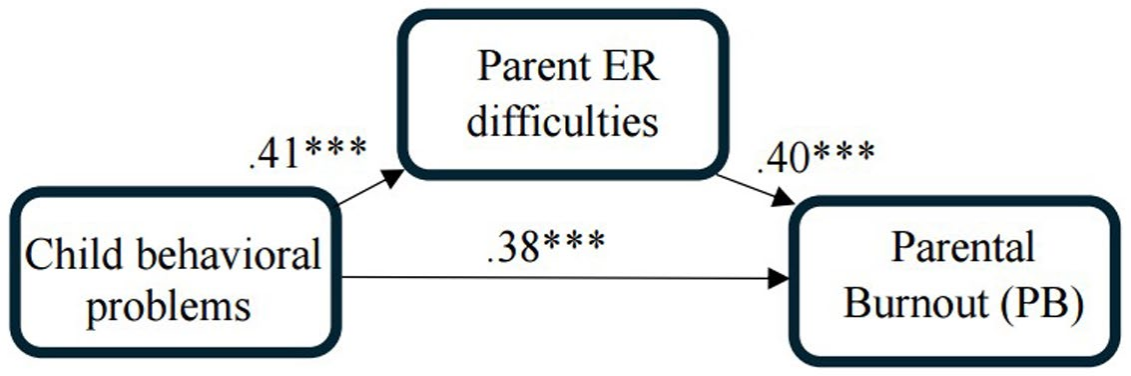
Standardized coefficients of the mediation model: parent ER difficulties mediate the effect of child behavioral problems on PB, while controlling for changes in the educational setting and the main caregiver of the child(ren) following the war as covariates.

The results indicated that the parent ER difficulties serve as a mediation variable between child behavioral problems and PB during the Swords of Iron war (the completely standardized indirect effect was *B* = 0.16, *SE* = 0.03, *LLCI*(95%) = 0.10, *ULCI*(95%) = 0.23). This result indicates that the higher the child’s behavioral problems are, the greater the difficulty their parents experience in the ER, which in turn leads to higher levels of PB (see Figure 1 for the standardized coefficients of the mediation model).

An explanation for the quantitative findings can be understood from the analysis of interviews conducted with parents of children with ASD.

### Qualitative results

In this study, 18 parents of children with ASD aged 6–21 were interviewed. The analysis of the interviews showed a trend that passed as the second thread among all the research participants and raises explanations for the quantitative results as described above.

The analysis of the interviews brought up three central issues in coping with a child with ASD during the war:

- Difficulty coping with the characteristics of the disability.
- Burnout in the role of parenting.
- Parental resilience.

All parents raised difficulties in coping with their child, especially during the war. Most of the difficulties that have arisen stem from the behaviors that are typical of the disabilities characterizing ASD and have been exacerbated by the stressful situation in which the State of Israel is. These behaviors led the parents to difficulty in regulating their emotions and sometimes even to a higher level of burnout in the role of parenting, as can be seen in the following examples:

*“…he does not speak and many times has tantrums…a very difficult feeling of helplessness. Sometimes the tantrum reaches physical violence towards me or towards his sister. I work on myself to be a model for him and show tolerance and flexibility to his behaviors. It does not always work out for me, but I really try and try…I’m very discouraged’…(interviewee 1)…”I feel like everything is going hard, the morning is hard, everything…I do not have too many conversations with him because he does not really communicate. Sometimes it’s really hard for me to see him like that, he does not understand and does not speak. It’s hard for me, it really hurts me… sometimes I go into such a black mirror. Everything is sad, everything is hard. But I continue the routine even though it’s hard for me” (Interviewee 2)…”He curses, he beats, messes up the house… There was an alarm now in the war. We did not send him to kindergarten either…his behaviors, it annoys me, it upsets me so we also fight a lot…not easy, not easy. It’s hard…I get very angry with him, I never know where the line is…”(interviewee 3)…”he can suddenly fall on the floor crying…there are many times when I am very angry with him. I look at things he does and I want to cry…sometimes I do not have the ability to continue listening many times I’m really frustrated and it’s hard for me. Lately there has been a deterioration in his behavior and I really take it seriously. Sometimes I cry next to him and raise my voice very, very loudly. I get to the days when I have had enough - I no longer have anywhere to draw strength from…” (interviewee 5)…” There are bedtime rituals. It takes an hour and a half to fall asleep. At four in the morning he woke up and wants to leave the house and if not, he screams. He′s mad at me because I’m in the reserves… there are capsules in the daycare, it breaks the routine. He had a hard time with these transitions… in the evening when you want to sleep he wants to play… the war caught me off guard… sometimes I yell at him… it’s hard for us as parents. A lot of helplessness and inability… a period of crisis. But I get up and do it…” (Interviewee 7)…”There are many objections. The little something is enough and he “breaks the rules “. Two days ago he really hit me. Must always travel the same way to kindergarten. It’s enough that I changed something small, he cries and rages…sometimes the tones rise…I would like to be a better mother, a little more inclusive and more patient…”(interviewee 9)…”the war made him anxious. Can swear, unkind words…it’s a daily struggle. I have to be around to make sure everything is done…meals…very very challenging. I have to be the one who directs a conversation, feels, separates. Very, very challenging…”(Interviewee 11)…”The difficulty in the war was the lack of routine and whole days at home…the war with the Zoom – wasn’t good…it was difficult, there was frustration. I did a workshop on emotion management and emotional regulation. If our behavior is regulated, the girl’s difficulties will be reduced…”(Interviewee 12)…”He has difficulty in speaking. It comes out in behavior, nerves, withdrawal, outbursts… Raising a child with low-functioning ASD is very complex. All the time to work in front of him, empower and promote. It affects his entire lifestyle and ours and everything is conducted according to it… we employed ten people… it took us a lot of mental resources… our entire life path is according to him…” (Interviewee 13).*

The interviews revealed that parents consistently reported intensified challenges related to their children’s ASD characteristics during wartime. These difficulties primarily manifested in their children’s resistance to altered routines, such as taking new travel routes after relocation, struggles with activity transitions, and increased episodes of sudden anger outbursts. While these behaviors are typical of ASD, parents emphasized that their intensity and frequency became markedly more severe following the outbreak of war.

In response to these escalated behaviors, parents found themselves responding in ways they considered uncharacteristic, including raising their voices more frequently and experiencing heightened frustration. As one parent *(Interviewee 13)* expressed, *“Our entire life path is dictated by him,”* reflecting a sense of losing control over household management. Parents consistently emphasized the substantial emotional and mental resources required to cope with these intensified challenges during the crisis period.

All the parents described a similar process, both in terms of the difficulty in coping with their child’s behavior, and in terms of their parental regulation. Most of the parents testify that despite the great complexity created at home, they are indeed worn out, tired and sometimes helpless, but they do not allow themselves to break down, as can be seen in the following examples:

*“…I manage to cope with difficulty, I have to do it for the sake of my children. I must not enter into these thoughts for their benefit…” (interviewee 1)…”but I continue the routine even though it’s difficult for me” (interviewee 2)…” a period of breakdown. But I get up and do…work on myself. The process is important to me…” (interviewee 7)…”it’s impossible for it to manage you all day…it drains my strength so I know what I’m standing for…”(interviewee 8)…”I have to act flexibly otherwise I’ll collapse…” (interviewee 9)…”my psychologist taught me to aspire to” Good enough parent…”there are three chicks, (little children), who see the world so I do not have much choice”…(interviewee 10)…”any period that has a challenge like the war, is forging. Both the Corona virus, the war, the whole situation is forging and gives inner strength”…(Interviewee 11)…”.My parenting of a daughter with special needs strengthens my ability to deal with extreme situations, to contain and see the event”… (Interviewee 12).*

These examples show the resilience that parents of children with ASD have and their ability to regulate their emotions without bringing themselves to complete burnout. The need to protect the family, to protect the other children in the family and in fact the understanding of each parent that he must create a reservoir of coping energies, for himself and for his family, makes him do different things to maintain his personal resilience. Some parents have chosen professional treatment, some have chosen to keep the routine, and some enrich these reserves of strength in ways that suit them. Some parents even see this situation as a defining event so that their coping with the extreme situation they found themselves in, forges them, gives them inner strength and will enable them to deal with extreme situations throughout their lives. This point of view is a source of strength for them in coping with their child with ASD during wartime.

## Discussion

Parents of children with ASD are especially susceptible to burnout due to the challenges associated with their children’s developmental difficulties (Sekułowicz et al., 2022). This vulnerability is exacerbated during times of armed conflict or war, posing even greater challenges for these parents (Yılmaz et al., 2021). The disruptions caused by wartime not only affect access to special education, therapies, and treatments but also disrupt established routines, which can significantly impact children with ASD, leading to heightened difficulties in adaptation and increased emotional and behavioral problems (White et al., 2021). These challenges, in turn, exacerbate the unprecedented and significant demands their parents face during this time and serve as strong predictors of PB (Holly et al., 2024; Kaba et al., 2023). Our research aligns with previous studies, indicating that as behavioral problems escalate, so does PB (Kaba et al., 2023; Kütük et al., 2021). Qualitative insights from parents further highlight how the exacerbation of their child’s behavior during wartime contributes to their feelings of fatigue, stress, and difficulty in managing familial relationships, all of which contribute to their experience of burnout.

Previous studies have found correlations between child behavioral problems and parents’ ER difficulties (Findling et al., 2023; Kütük et al., 2021; Prikhidko and Swank, 2019), as well as between parents’ ER and PB (Brandão et al., 2024; Santelices et al., 2022). Our results are consistent with these prior findings, indicating positive correlations between child behavioral problems, parents’ ER difficulties, and PB. According to the BR2 theory, these associations can elucidate the parental burnout observed among parents of children with ASD during the war. The compounding risk factors they encounter—such as the heightened stress of parenting a child with ASD, the exacerbated behavioral problems of their children and consequent ER difficulties during wartime—may exacerbate the imbalance between parental risks and resources, consequently leading to parental burnout.

Previous studies have demonstrated the association between child behavioral problems and PB with ER, which supports the notion of parent ER acting as a mediator between these factors (Brandão et al., 2024; Prikhidko and Swank, 2019; Santelices et al., 2022). Additionally, these studies indicate that parent ER may function as a moderating variable, contributing to the reduction of PB (Prikhidko et al., 2020; Swit and Breen, 2023; Vertsberger et al., 2022). In our study, we observed that parents’ ER difficulties mediated the relationship between child behavioral problems and PB.

In alignment with the mediation model identified by Qiao et al. (2024), our findings also indicate that emotion regulation serves as a pivotal factor in the relationship between child behavioral problems and their implications for parental functioning. However, in contrast to their study, which focused on the child’s emotional regulation, our investigation examined parental emotion regulation. The similarity in these findings reinforces the close connection and bidirectional influence between child and parental emotion regulation, as previously established by Hajal and Paley (2020). Furthermore, this mediation model suggests that parental emotion regulation may function as a mechanism through which children’s behavioral challenges impact parental outcomes. This conceptualization aligns with the growing body of literature emphasizing the importance of considering emotion regulation as a family-level process, encompassing multiple subsystems within the family unit (Paley and Hajal, 2022).

Our study examined the impact of socio-demographic factors and other background changes caused by the Swords of Iron war on child behavioral problems, parental ER difficulties, and PB. We found that changes in family routines played a significant role in explaining these issues. Mikolajczak and Roskam (2018) argue that socio-demographic factors did not contribute additional variance in PB beyond the impact of the other risk and resource factors encompassed in their original balance. However, the specific factors of having a child with a disability or chronic illness and being in a wartime context were not included in the balance and have not been tested under this model so far. Specifically, the change in the educational setting emerged as the strongest predictor of the child’s behavioral problems, the parent’s ER difficulties, and PB. Special education schools not only provide full-day childcare and educational services but also offer healthcare support, various therapies, and additional adapted services and supports. Therefore, disruptions to schools become a critical additional stressor for families with a child with ASD during an already stressful time due to the war. This finding is consistent with previous research demonstrating that during COVID-19, changes in routine and disruptions to children’s special education led to serious behavioral problems in children with ASD, increased parental stress levels, difficulties in maintaining their mental well-being, and a negative impact on emotion management (Yılmaz et al., 2021). Our qualitative data also indicate that parents highlighted how changes in the educational setting during the war significantly affected their child’s behavior, worsened ASD symptoms, and contributed to a stressful atmosphere at home.

An interesting finding that emerged from our study is that ER did not moderate the correlation between children’s behavioral problems and PB among the Israeli parents of children with ASD during the war. While in other contexts, ER has been recognized as a protective factor against PB (Brandão et al., 2024), our research suggests that ER alone might not be adequate to alleviate the impact of wartime demands on parents of children with ASD. Various explanations may elucidate this finding.

First, in our study, we assessed parents’ levels of ER difficulties, which were revealed to be medium-low (mean score of 2.45 on the range of 1–5). It’s important to note that having low ER difficulties does not necessarily equate to possessing strong ER skills, as highlighted by Mikolajczak and Roskam (2018) that resources are not the absence of risks. This could explain why parents with low ER difficulties may still experience high levels of burnout, as their ER skills might not be sufficient, meaning their ER does not play the part of a protection factor. While we discovered that the absence of ER difficulties alone is not adequate to decrease PB, we did find a positive correlation between ER difficulties and PB. This suggests that ER may be effective in reducing PB when combined with additional resources.

An additional explanation for our findings may be the exacerbate imbalance between risks and resources during the specific context of Swords of Iron war. Based on our research findings, the specific period of wartime presents a unique context that may alter the predicted risk/resource balance as suggested by the BR2 theory. While this imbalance is a common phenomenon to all burned-out parents, the specific risks and resources vary for each individual, leading to unique experiences of burnout, especially in extreme situations such as wartime (Mikolajczak and Roskam, 2018). Individual differences in mental health and cognitive processes play a significant role in how emotions are regulated. For instance, acute stress can hinder the ability to employ effective ER strategies (Prikhidko and Swank, 2019), and cognitive factors, such as the need for a high degree of control, can impact the effectiveness of ER strategies during stressful situations (Holly et al., 2024). In the demanding context of parenting a child with ASD during wartime, the interplay between mental health and cognitive processes may be even more pronounced, suggesting that other psychological variables could mitigate the potential benefit of lacking ER difficulties. The insights shared by participants in the interviews revealed that parents described their own protection factors, enabling them to regulate their emotions according to their subjective perception. The desire to protect their family members, coupled with the capacity to replenish their strength through various personalized means, serves as a protective factor for parents, even amidst the considerable stress of wartime and the behavioral changes observed in their child with ASD.

Our research shows that more work is needed to be capable of explaining why parents of children with ASD burned out, what is the contribution of the specific time of crisis to PB, and what are the available resources that parents of children with ASD can use in this challenging time. Our findings highlight the significant impact of changes in education setting on child’s behavioral problems associated with parent higher ER difficulties and PB during wartime. These relationships may help predicting who is at higher risk for PB in these extraordinary circumstances, necessitating prioritization of services for children with ASD and their parents.

Notwithstanding that this study was conducted on a specific population during a particular emergency period, it is possible to extrapolate and generalize these conclusions to other populations and diverse emergency situations. Even though there may be variations in familial mechanisms, interpersonal relationships, and intrapersonal processes across different cultures, populations, and individuals, one can still identify commonalities in the conditions and ramifications of various emergency situations on people. Consequently, the findings of this research may contribute to a broader understanding of parental burnout and emotional regulation among parents in general.

### Limitations and recommendations for future research

The present study has several limitations that warrant consideration. The participants were recruited through convenience sampling, which might not adequately represent the population. In addition, during the Swords of Iron war, certain areas in Israel, both in the north and in the south, were evacuated from their homes. In the scope of this study, a distinction was not made between evacuees and non-evacuees; rather, parents were asked only if there was a change in their residence in general. In further research, it is important to consider the evacuation experience of families with children with disabilities in general, and ASD in particular. Additionally, the study did not examine the socio-economic status of the parents, despite previous research indicating a negative correlation with PB (Kuru and Piyal, 2018; Lin et al., 2023). Therefore, further research should be conducted with larger samples and include populations from all regions of the country. Utilizing random sampling methods would improve the representativeness of the sample and mitigate potential biases.

An important limitation of the study is its use of a cross-sectional design, which precludes making causal inferences. Additionally, the absence of a comparison group of parents of children with ASD during routine times, as opposed to wartime conditions, limits the ability to assess the specific impact of wartime on the variables under investigation. As previously mentioned, this unusual context may introduce, alter, or remove factors included in the BR2 theory, or change their relative importance. To address these limitations and advance the field, future research could employ longitudinal designs to provide a more comprehensive understanding of the relationships between children’s behavioral changes, parents’ ER strategies, and PB during wartime. Similarly, the absence of a comparison group of parents of children without ASD limits the ability to evaluate the specific impact of the child’s ASD on the variables under investigation. The BR2 assessment tool lacks the specific risk factor of having a child with ASD, which is a significant circumstance to consider when using this assessment. Future research should investigate the overall effect of having a disabled child on the presence, absence, level, or weight of other risk and resource factors by comparing parents of children with and without ASD.

Finally, our study only examined the children’s irritability and hyperactivity/noncompliance subscales from the Aberrant Behavior Checklist, based on previous studies indicating their significant influence on PB and well-being (Kaat et al., 2014; Kaba et al., 2023). However, from our interviews with the parents, it became apparent that another significant factor affecting their ER and subsequently their burnout was the exaggerated ASD stereotypical behaviors of their child during the war. It is possible that the extreme circumstances of wartime have heightened the influence of children’s stereotypic behavior, leading this component to have a stronger impact on parents during this specific time. Future research should explore other aspects of behavioral problems and ASD symptoms to provide a more comprehensive understanding of the relationship between wartime, children’s behavioral alterations, and their impact on parents’ well-being and other outcomes.

### Implications for practice

Individuals with ASD and their families are an important vulnerable group for consideration of adapted services and additional support during emergency situations. Therefore, parents of children with ASD worth intensive monitoring by clinicians and other service providers as well as attempts to develop and share resources that may increase coping and reduce stress. Interventions aimed at mitigating PB via ER should also apply to other factors such as rebalancing the family and professional lives, devoting more time for leisure, searching for practical and emotional social support, improving childrearing practices, improving the relationship and communication with the coparent, or working on the need for control, perfectionism, and self-efficacy beliefs (Mikolajczak and Roskam, 2018). Specifically, it is important to consider all aspects of parents’ psychological context and put special emphasis on decreasing stress, as acute stress limits the ability to use effective ER strategies. Interventions designed specifically to parents of children with ASD should be considering their special circumstances and put extra emphasis on the children’s behavioral problems. Finally, additional interventions that can be adapted and delivered remotely to children with ASD and their parents during emergency times should be developed.

## Data Availability

The raw data supporting the conclusions of this article will be made available by the authors, without undue reservation.
